# Letter to the editor: Prevention of bacterial sexually transmitted infections (STI) in France: why not recommend using condoms and safer sex?

**DOI:** 10.2807/1560-7917.ES.2019.24.12.1900171

**Published:** 2019-03-21

**Authors:** Eric Caumes

**Affiliations:** 1 Sorbonne Université, AP-HP, Hôpitaux Universitaires Pitié-Salpêtrière Charles Foix, Service de Maladies infectieuses et Tropicales, Paris, France; 2 Sorbonne Université, INSERM, Institut Pierre Louis d’Épidémiologie et de Santé Publique, Paris, France

**Keywords:** Sexually transmitted infections, prevention, safer sex, condoms, France

**To the editor:** I read with great interest the article by Ndeikoundam Ngangro et al. entitled ‘Bacterial sexually transmitted infections (STI) in France: recent trends and patients’ characteristics in 2016’, which showed an increased number of cases of syphilis, rectal lymphogranuloma venereum (LGV) and gonorrhoea in France between 2014 and 2016 [1]. The recommendation of the authors is ‘regular screening of patients and partners followed by prompt treatment to interrupt STI transmission’. Interestingly, the words ‘safer sex’ and ‘condom’ are not stated in the sections focussing on prevention in the article. The authors underline that ‘HIV prevention has expanded towards medical prophylaxis’ but this does not concern STI prevention, as medical prevention of HIV infection with pre-exposure prophylaxis (PrEP) does not protect against other STI and must thus be associated with condom use.

The reason why French public health specialists only recommend the test and treat approach for STI instead of prevention needs to be better explained; the continuous and marked surge of gonorrhoea and rectal LGV in men that have sex with men (MSM), for example, likely warrants more than a test and treat approach. There are at least eight reasons to worry about this continuous increase of STI: (i) re-occurrence of severe complications of gonorrhoea and syphilis with high rate of sequelae, (ii) worldwide increase of resistance to antibiotics including STI agents as recently illustrated with extensively drug-resistant (XDR) *N.gonorrhoeae* and XDR *Mycoplasma genitalium*, (iii) increase in the number of sexual partners per year among MSM, (iv) gastrointestinal (GI) and liver diseases related to the faecal-oral route of transmission particularly among MSM, (v) appearance of blood-borne diseases such as hepatitis C in the subgroup of highly sexually active MSM, (vi) discovery of possible sexual route of transmission for emerging infectious diseases (Ebola and Zika virus disease, Rift valley fever etc.), (vii) possibility for these latter diseases to be transmitted months after cure and (viii) history as story repeats itself [2].

Is it possible that sexually transmitted disease history, including AIDS history, has been largely forgotten? From a historical perspective, AIDS epidemics in MSM were preceded by an increase in the number of sexual partners, STI diagnoses (such as gonorrhoea and syphilis) and outbreaks of faecal-orally transmitted GI and liver diseases [2]. On another hand, safer sex and condom use were the main tools for tackling the AIDS epidemic before antiretroviral treatments were largely available [3].

With a continuous increase in STI, it is surprising that condom use and safer sex is not more actively promoted by public health authorities [1]. A major role of public health and infectious disease specialists should be to recall the general rules of STI prevention, i.e. condom use and safer sex—whether associated with biomedical prophylaxis or not, as the latter also carries the risk of antimicrobial resistance.

Indeed, promoting the repeated use of antibiotics for treatment of recurrent STI and the use of PrEP for prophylaxis of HIV infection is questionable, as condoms are an effective and harmless prevention tool. Moreover, this contrasts with the current recommendations for controlled use of antimicrobial therapy to lower antimicrobial resistance [4].

In conclusion, it should be highlighted that prevention of STI, through condom use and safer sex, is better, less harmful and cheaper than a cure [5]. This is particularly important with ever increasing concerns about antibiotic and antiretroviral resistance in a population where STI are increasing.
